# Influenza A Subtype H3 Viruses in Feral Swine, United States, 2011–2012

**DOI:** 10.3201/eid2005.131578

**Published:** 2014-05

**Authors:** Zhixin Feng, John A. Baroch, Li-Ping Long, Yifei Xu, Frederick L. Cunningham, Kerri Pedersen, Mark W. Lutman, Brandon S. Schmit, Andrew S. Bowman, Thomas J. DeLiberto, Xiu-Feng Wan

**Affiliations:** Mississippi State University, Mississippi State, Mississippi, USA (Z. Feng, L.-P. Long, Y. Xu, X.-F. Wan);; Jiangsu Academy of Agricultural Sciences, Nanjing, China (Z. Feng);; Animal and Plant Health Inspection Service of the US Department of Agriculture, Fort Collins, Colorado, USA (J.A. Baroch, K. Pedersen, M.W. Lutman, B.S. Schmit, T.J. DeLiberto);; Animal and Plant Health Inspection Service of the US Department of Agriculture, Mississippi State (F.L. Cunningham);; The Ohio State University, Columbus, Ohio, USA (A.S. Bowman)

**Keywords:** feral swine, H3, influenza A virus, swine influenza virus, serologic assay, seropositive, H3N2v, viruses, United States

## Abstract

To determine whether, and to what extent, influenza A subtype H3 viruses were present in feral swine in the United States, we conducted serologic and virologic surveillance during October 2011–September 2012. These animals were periodically exposed to and infected with A(H3N2) viruses, suggesting they may threaten human and animal health.

Swine are proposed as “mixing vessels” to generate novel influenza A viruses (IAVs) by facilitating reassortment among IAVs and providing a potential pathway in which these viruses can move from wild birds to humans (*1*). Subtype H3N2 is one of the most common subtypes in the US domestic swine population, which possibly resulted from spillover of human seasonal A(H3N2) virus (*2*,*3*). Since its introduction in the mid-1990s, A(H3N2) virus has evolved genetically and antigenically in domestic swine. Four genetic groups (so-called clusters I–IV) were identified, and these 4 clusters were also antigenically distinct (*4*). The viruses in cluster IV formed at least 2 antigenic subclusters, H3N2-α and H3N2-β (*5*). Both subclusters are co-circulating in pigs, and subcluster H3N2-β predominated among the isolates obtained from domestic swine at Ohio county fairs during 2010–2011 (*5*). During July and August 2011, two children were infected with novel reassortant H3N2 variant (H3N2v), 1 in Indiana and 1 in Pennsylvania (*6*). This H3N2v virus is antigenically similar to the viruses in subcluster H3N2-β and has the matrix gene of influenza A(H1N1)pdm09 virus. It caused illness in ≈2,055 persons during August 2011–April 2012 (*7*).

The role of feral swine in IAV ecology has not been adequately addressed. Feral swine could be a reservoir of IAVs or, possibly, a spatially dynamic mixing vessel, given their free-ranging habits. Such unrestricted movement provides the potential for exchange of IAVs among subpopulations of feral swine and the opportunity for exposure to different IAVs through contact with a variety of habitats and species. Also, feral swine can live up to 8 years, which provides ample opportunities for reinfection with the same subtype IAVs, especially those with antigenically distinct hemagglutinins. The IAVs can be transmitted bidirectionally between feral and domestic swine because contact between them is not unusual (*8*). Ultimately the IAVs emerging in feral swine potentially could be transmitted to humans.

The United States has ≈4–5 million feral swine (*9*) throughout at least 38 states (*10*). Feral swine are expanding their range because of a lack of natural predators and intentional introductions for hunting. Our goals in this study were to determine through virologic and serologic surveillance whether, and to what extent, subtype H3 IAVs were present in the US feral swine metapopulation.

## The Study

We collected 1,983 nasal swab samples from swine during October 2011–September 2012 (Technical Appendix). Matrix gene–based quantitative reverse transcription PCR showed that 9 swabs samples were IAV positive; 1 A(H3N2) feral swine isolate, A/swine/Texas/A01104013/2012(H3N2), was recovered. Phylogenetic analyses showed that all genes of this feral swine isolate are genetically similar to those of A(H3N2v) viruses isolated from humans, and other contemporary subtype H3N2 isolates from swine from county fairs farms. The matrix gene of this feral swine IAV was genetically close to that of A(H1N1)pdm09 and the human A(H3N2v) viruses (Technical Appendix) (*11*). Similar to other viruses in the antigenic cluster, H3N2-β A/swine/Texas/A01104013/2012(H3N2) had a R189K mutation at the antibody-binding site of the hemagglutinin protein, which caused a recent antigenic drift in subtype H3N2 IAVs (*5*,*12*). The 8 genes of A/swine/Texas/A01104013/2012(H3N2) have a minimal 99.59% nt sequence identity to those of the human subtype H3N2v isolate A/Indiana/10/2011(H3N2).

**Figure 1:**
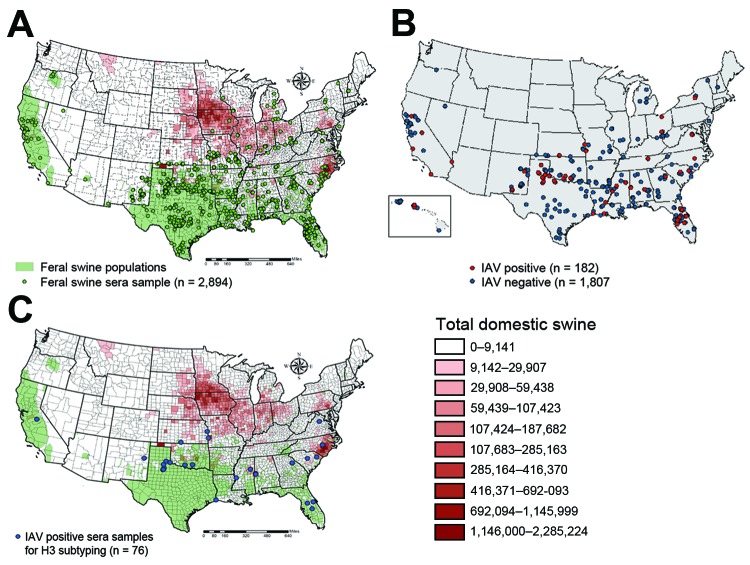
Geographic distributions of seum samples from feral swine, United States, 2011–2012. A) Of 1,989 samples tested by ELISA, 182 were positive (red) and 1,807 were negative (blue). B) The 76 samples (blue) were selected for hemagglutination-inhibition and microneutralization subtyping. C) The distributions of feral swine (green) and domestic swine (orange) were also marked (A and C).

**Figure 2:**
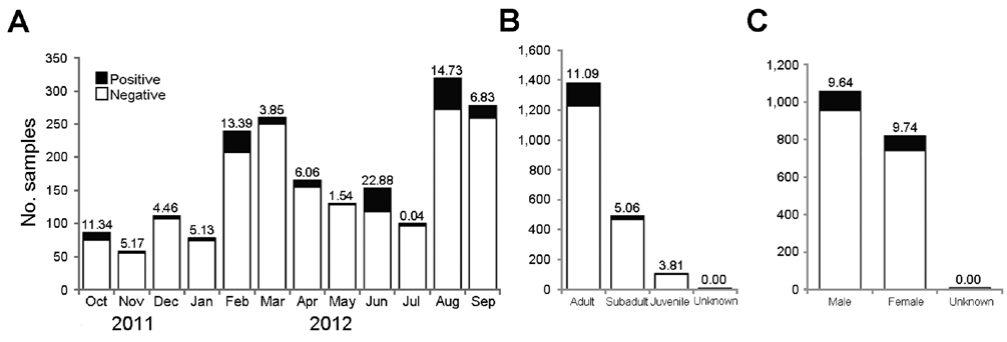
Epidemiologic analyses of feral swine serum samples seropositive for influenza A virus by ELISA, United States, 2011–2012. A) Temporal distribution. B) Distribution of feral pigs, by age. C) Distribution of feral pigs, by sex. The numbers on the bar indicate the influenza A virus–seropositive percentile.

We also collected 1,989 serum samples from swine in 31 states; these samples were tested by using an IAV-specific ELISA (Figure 1). We identified 182 samples as IAV positive, from swine that were broadly distributed over 19 states. The average IAV seropositive rate was 9.15% but it varied by month. The highest positive rate (22.9%) was in June 2012 (Figure 2). Although no clear temporal pattern was found in the IAV seropositive rate, the rate was relatively higher in the summer than in other seasons. One explanation could be that noncommercial swine farmers might give their animals more pasture time during the summer, thereby increasing the chance of contact between domestic and feral swine. In addition, our results showed adult feral swine (>1 year of age) had the highest rate of IAV positivity (11.1% [of 1,380 animals]), followed by subadults (2 months–1 year of age) (5.1% [of 494 animals]), and juveniles (<2 months of age) (3.8% [of 105 animals]) (age was not determined for 10 animals). (Dentition patterns were used to determine the age of feral swine [*13*].) Female and male pigs were equally as likely to be seropositive (102 [9.6%] of 1,058 vs. 80 [9.7%] of 821).

Of the 182 IAV-positive serum samples, 76 were randomly selected for influenza subtyping. We used hemagglutination-inhibition (HI) and microneutralization (MN) assays for subtyping against A(H1N1)pdm09 virus and 22 H3 IAVs, which represent a wide range of antigenically distinct H3 IAVs (Technical Appendix). Serum was defined as seropositive if its titer was >40.

HI results showed that 46 (60.5%) of 76 feral swine samples were positive against at least 1 of the 22 H3 IAVs tested, of which A/swine/Texas/A011040013/2012(H3N2) had the highest seropositive rate (47.4%), followed by 4 human A(H3N2v) isolates (Table). MN results were consistent with those of HI. The geometric mean titers for HI and MN against the feral swine isolate were 163 and 259, respectively. The maximum MN titer among the serum and A/swine/Texas/A011040013/2012(H3N2) was 1,280. The seropositive rates of these serum samples varied from 34.2% to 42.1% against the other viruses from subclusters H3N2-α and H3N2-β (Table), but the viruses in subcluster H3N2-β had significantly higher geometric mean titers than did those in H3N2-α (p<0.001).

**Table:** Cross-reactive antibody responses against H3 and influenza A(H1N1)pdm09 virus in 76 influenza-positive serum samples from feral swine, United States, 2011–2012*

Source, virus	Antigenic cluster†	No. seropositive (%)‡			GMT (95% CI)§		No. overall seropositive (%)¶
		HI	MN		HI	MN	
Feral swine IAV							
A/swine/Texas/A011040013/2012(H3N2)	H3N2-β	36 (47.4)	36 (47.4)		163 (40–640)	259 (40–1,280)	25 (32.9)
Domestic swine IAV							
A/swine/Ohio/11SW64/2009 (H3N2)	H3N2-α	27 (35.5)	30 (39.5)		65 (40–160)	121 (40–1,280)	17 (22.4)
A/swine/Iowa/12627/2009(H3N2)	H3N2-α	30 (39.5)	31 (40.8)		73 (40–320)	183 (40–1,280)	15 (19.7)
A/swine/Ohio/11SW347/2011(H3N2)	H3N2-β	31 (40.8)	31 (40.8)		122 (40–640)	112 (40–1,280)	17 (22.4)
A/swine/Iowa/6368/2012(H3N2)	H3N2-β	26 (34.2)	36 (47.4)		94 (0–640)	240 (40–1,280)	20 (26.3)
Human H3N2v IAV							
A/IA/07/2011		32 (42.1)	31 (40.8)		109 (40–640)	313 (40–1,280)	21 (27.6)
Human seasonal IAV	H3N2-β						
A/Perth/16/2009	H3N2-β	12 (15.8)	5 (6.6)		76 (40–320]	121 (40–1,280)	1 (1.3)
A/Victoria/361/2011	H3N2-β	11 (14.5)	2 (2.6)		141 (40–640)	226 (40–1,280)	0
A/California/7/2009	H3N2-β	5 (6.6)	3 (3.9)		70 (40–640)	80 (40–320)	1 (1.3)

*Influenza detected by ELISA. GMT, geometric mean titer; HI, hemagglutination-inhibition; MN, microneutralization; IAV, influenza A virus. †The antigenic cluster H3N2-α and H3N2-β were defined in (*5*). ‡Seropositive was defined as the HI or MN titer >40. §Only serum with an HI or MN titer >40 are used to calculate the GMT. ¶Overall seroconversion was defined as both HI and MN titer >40.

HI results also showed that 12 samples were seropositive against A/Perth/16/2009(H3N2); 11 were seropositive against A/Victoria/361/2011(H3N2), and 5 were seropositive against A/California/7/2009(H1N1). These 3 viruses do not cross-react with the IAVs from subclusters H3N2-α and H3N2-β. Those 5 subtype H1N1-positive samples were also seropositive against subtype H3N2 IAVs, indicating potential previous exposures of these feral swine to both H3 and H1 IAVs.

The HI results demonstrated that only 2 serum samples had a low-level cross-reaction with avian influenza A(H3N2) viruses, and the HI titers for both were 40. This result is consistent with findings in an earlier report (*14*). Further studies are needed to determine whether additional antigenic clusters of H3 IAVs are present in migratory waterfowl. The discrepancies in the cross-reactivity of these serum samples against the IAVs tested in this study suggested that these feral swine had different exposure histories against antigenically diverse IAVs.

## Conclusions

Our study demonstrated that subtype H3N2 IAVs are periodically infecting feral swine in the United States. Feral swine are a potential source of IAVs with bidirectional transmission to domestic swine or humans. Detection of an H3N2v-like IAV in the feral swine population demonstrates a potential threat to human health. Continued surveillance is recommended to monitor the distribution and the genomic and antigenic diversities of IAVs in feral swine to better assess the risk.

Technical Appendix Detailed materials and methods and phylogenetic analysis of various gene segments of influenza A(H3N2) virus recovered from feral swine, United States, 2011–2012.
