# Supplementary material for: Influenza A Subtype H3 Viruses in Feral Swine, United States, 2011–2012
Source: Emerg Infect Dis. 2014 May;20(5):843–6. doi: 10.3201/eid2005.131578 (PMC4012812; doi:10.3201/eid2005.131578)
Supplement: Technical Appendix — Detailed materials and methods and phylogenetic analysis of various gene segments of influenza A(H3N2) virus recovered from feral swine, United States, 2011–2012. [file 13-1578-Techapp-s1.pdf]

Article DOI: http://dx.doi.org/10.3201/eid2005.131578

Influenza A Subtype H3 Viruses in Feral Swine, United States, 2011–2012

Technical Appendix

Material and Methods

Sample Collection

Samples were collected opportunistically from feral swine removed for wildlife damage management purposes. Blood samples were collected primarily by cardiac puncture and placed in serum-separating collection tubes. Once the blood was clotted, it was centrifuged, and the serum was transferred into 2-mL cryogenic vials and labeled with a unique barcode. Serum samples were shipped to the US Department of Agriculture (USDA), National Wildlife Disease Program (Fort Collins, CO, USA), where they were stored frozen at –80°C until testing. Nasal swabs were collected by inserting a sterile Dacron swab in the nasal cavity and gently swabbing the surface of the nasal mucosa. This process was repeated in both nostrils with the same swab, placed in a cryogenic vial with 3 mL of brain-heart infusion broth provided by the USDA National Veterinary Services Laboratories (NVSL), and the vial was labeled with a unique barcode. Vials were kept in a cooler with ice packs until they were shipped to the testing laboratory within 3 days after collection.

Viral Isolation and Subtyping

The nasal swabs from feral swine were screened by using influenza A virus (IAV) matrix gene based quantitative reverse transcription PCR at 1 of the National Animal Health Laboratory Network facilities across the United States (*1*). Matrix-positive samples were tested with an N1 subtyping PCR. Matrix-positive results were forwarded to NVSL for testing regardless of the N1 subtyping result. Isolates were recovered by using 9- to 11-day-old specific pathogen-free (SPF) eggs according to the protocols described in the Swine Influenza Surveillance Procedure Manual (www.aphis.usda.gov/animal_health/animal_dis_spec/swine/siv_surveillance.shtml). The

hemagglutinin (HA) and neuraminidase (NA) subtypes were determined at the NVSL using standard hemagglutination-inhibition (HI) and NA inhibition testing procedures.

Viruses

A total of 22 H3 IAVs were used for comparison in serologic assays. In addition to the feral swine isolate A/swine/Texas/A01104013/2012(H3N2) recovered from this study, 4 human H3N2v isolates, 3 county fair pig H3N2 isolates, 7 domestic pig H3N2 isolates, 2 migratory bird H3N2 isolates, 2 canine H3 isolates, 2 human seasonal H3N2 isolates, and 1 2009 H1N1 isolate were used in the serologic assays (Technical Appendix Table).

To maximize the chances of detecting H3 IAV infection in feral swine samples tested, the 22 isolates described above were selected to represent a wide range of antigenically distinct H3 IAVs (Technical Appendix Table). Ten of them represented 2 of the contemporary H3 antigenic clusters in the swine population: H3N2-α and H3N2-β (*2*). Four human H3N2v isolates were also included, and these isolates antigenically belong to H3N2-β.

A/Perth/16/2009(H3N2) and A/Victoria/361/2011(H3N2), 2 seasonal influenza vaccine strains recommended by the World Health Organization (WHO), represent the antigenic variants predominantly circulating in human population. These viruses do not cross-react with the 10 swine IAVs and 4 H3N2v viruses described in the previous paragraph. An avian origin H3N2 canine influenza virus (CIV) (*3*) and an equine origin H3N8 CIV (*4*) were also selected, which do not cross-react with swine H3N2, human H3N2v, or H3N2 seasonal influenza viruses. Except for H3N2 CIV, the 2 H3 avian influenza viruses selected do not cross-react with the other isolates listed in the Table. The selected 2009 H1N1 virus also did not react with any H3 IAVs selected for this study (Technical Appendix Table).

All avian influenza viruses were propagated in MDCK cells (ATCC, Manassas, VA, USA) and then stored at −70°C until analysis.

Serologic Assays

Influenza-specific ELISAs were performed by using IDEXX Influenza A Ab Test (IDEXX, [Westbrook, ME, USA) based on the manufacturer’s specifications. HI assays were performed according to the WHO manual on animal influenza diagnosis and surveillances (http://whqlibdoc.who.int/hq/2002/WHO_CDS_CSR_NCS_2002.5.pdf). Before HI tests were conducted, feral swine serum was treated with receptor-destroying enzyme (Denka Seiken Co.,

Tokyo, Japan) by 1:3 (v/v) at 37°C for 18 h, and then heat inactivated at 55°C for 30 min. Then, the serum was diluted with phosphate-buffered saline (for a final dilution of 1:10), and 22 influenza A viruses were tested by HI assay with 0.5% turkey red blood cells. The HI for the A/Victoria/369/2011(H3N2) testing was performed using 0.5% guinea pig red blood cells.

Microneutralization (MN) assay was performed in MDCK cells. Neutralizing titers were expressed as the reciprocal of the serum dilution that inhibited 50% of the viral growth of 100 tissue culture infectious doses of the virus. The MN titers were determined by HA assay using 0.5% turkey red blood cells as described (*2*).

Serologic Data Analyses

If the titers of a serum against a specific influenza isolate were ≥40, the serum was classified as positive to this isolate. Student *t* tests were performed to test the null hypothesis: there was no significant difference among the HI or MN titers between different groups of IAVs.

Molecular Characterization and Phylogenetic Analyses

The multiple sequence alignments were conducted by using the MUSCLE software package (*5*). The phylogenetic analyses were performed by using maximum-likelihood by GARLI version (*6*), and bootstrap resampling analyses were conducted with 1,000 runs using PAUP* 4.0 Beta (*7*) with a neighbor-joining method, as described (*8*).

References

1. Spackman E, Senne DA, Bulaga LL, Myers TJ, Perdue ML, Garber LP, et al. Development of real-time RT-PCR for the detection of avian influenza virus. Avian Dis. 2003;47(Suppl):1079–82. PubMed http://dx.doi.org/10.1637/0005-2086-47.s3.1079

2. Feng Z, Gomez J, Bowman AS, Ye J, Long LP, Nelson SW, et al. Antigenic characterization of H3N2 influenza A viruses from Ohio agricultural fairs. J Virol. 2013;87:7655–67. PubMed http://dx.doi.org/10.1128/JVI.00804-13

3. Li S, Shi Z, Jiao P, Zhang G, Zhong Z, Tian W, et al. Avian-origin H3N2 canine influenza A viruses in southern China. Infect Genet Evol. 2010;10:1286–8. PubMed http://dx.doi.org/10.1016/j.meegid.2010.08.010

4. Crawford PC, Dubovi EJ, Castleman WL, Stephenson I, Gibbs EP, Chen L, et al. Transmission of equine influenza virus to dogs. Science. 2005;310:482–5. PubMed http://dx.doi.org/10.1126/science.1117950

5. Edgar RC. MUSCLE: multiple sequence alignment with high accuracy and high throughput. Nucleic Acids Res. 2004;32:1792–7. PubMed http://dx.doi.org/10.1093/nar/gkh340

6. Zwickl DJ. Genetic algorithm approaches for the phylogenetic analysis of large biological sequence datasets under the maximum likelihood criterion. Austin (TX): The University of Texas; 2006.

7. Swofford DL. PAUP*: Phylogenic analysis using parsimony. Sunderland (MA): Sinauer; 1998.

8. Wan XF, Nguyen T, Davis CT, Smith CB, Zhao ZM, Carrel M, et al. Evolution of highly pathogenic H5N1 avian influenza viruses in Vietnam between 2001 and 2007. PLoS ONE. 2008;3:e3462. PubMed http://dx.doi.org/10.1371/journal.pone.0003462

Technical Appendix Table. Cross-reactive antibody responses among influenza A viruses used in this study by using HI assay against ferret antiserum for H3 influenza A virus and A(H1N1)pdm09 virus*

		Ferret antiserum‡									
	Antigenic	Swine influenza virus			Canine influenza virus		Avian influenza virus		Human seasonal influenza virus		
Virus†	cluster	09SW64	10SW215	11SW347	CIVH3N2	CIVH3N8	99AIVH3N2	11AIVH3N2	Perth09	Victora11	Pdm09
Isolates from agricultural fairs											
A/swine/Ohio/09SW64/2009(H3N2)	H3N2-α	**1,600**	40	<10	<10	<10	<10	<10	<10	<10	ND
A/swine/Ohio/10SW215/2010(H3N2)	H3N2-β	80	**960**	640	<10	<10	<10	<10	<10	<10	ND
A/swine/Ohio/11SW347/2011(H3N2)	H3N2-β	20	320	**576**	<10	<10	<10	<10	<10	<10	ND
Isolates from canine											
A/canine/Guangdong/1/2006(H3N2)		<10	<10	<10	**1,280**	40	320	160	ND	ND	ND
A/canine/Iowa/13628/2005(H3N8)		<10	<10	<10	40	**160**	<10	<10	ND	ND	ND
Isolates from avian											
A/blue-winged teal/Ohio/99–31/99(H3N2)		<10	<10	<10	ND	<10	**70**	20	ND	ND	ND
A/blue-winged teal/Ohio/11OS2474/2011(H3N2)		<10	<10	<10	ND	80	20	**320**	ND	ND	ND
Isolates from vaccine strains											
A/Perth/16/2009(H3N2)		<10	<10	<10	ND	ND	ND	ND	**40**	80	ND
A/Victoria/361/2011(H3N2)		<10	<10	<10	ND	ND	ND	ND	<10	**1,280**	ND
A/California/7/2009(H1N1)		<10	<10	<10	ND	ND	ND	ND	<10	<10	ND
Isolates from feral hog											
A/swine/Texas/A01104013/2012(H3N2)		40	>640	320	<10	<10	<10	<10	ND	ND	ND
Isolates from swine farms											
A/swine/Nebraska/9330/2006(H3N2)	H3N2-α	320	80	<10	<10	<10	<10	<10	ND	ND	ND
A/swine/North Carolina/1026/2007(H3N2)	H3N2-α	640	80	<10	<10	<10	<10	<10	ND	ND	ND
A/swine/Iowa/18469/2008(H3N2)	H3N2-α	320	<10	<10	<10	<10	<10	<10	ND	ND	ND
A/swine/Wisconsin/12627/2009(H3N2)	H3N2-α	1,280	80	80	<10	<10	<10	<10	ND	ND	ND
A/swine/Michigan/33261/2010(H3N2)	H3N2-β	160	1,280	1,280	<10	<10	<10	<10	ND	ND	ND
A/swine/Indiana/9622/2011(H3N2)	H3N2-β	40	1,280	1,280	<10	<10	<10	<10	ND	ND	ND
A/swine/North Carolina/6368/2012(H3N2)	H3N2-β	320	1280	640	<10	<10	<10	<10	ND	ND	ND
Isolates from patients											
A/Wisconsin/12/2010(H3N2)	H3N2-β	80	640	320	<10	<10	<10	<10	ND	ND	ND
A/Pennsylvania/14/2010(H3N2)	H3N2-β	320	640	640	<10	<10	<10	<10	ND	ND	ND
A/Minnesota/11/2010(H3N2)	H3N2-β	20	320	160	<10	<10	<10	<10	ND	ND	ND
A/Iowa/07/2011(H3N2)	H3N2-β	80	160	320	<10	<10	<10	<10	ND	ND	ND
Isolates from equine											
A/equine/Miami/1/63(H3N8)		<10	<10	<10	<10	<10	<10	<10	ND	ND	ND

*HI, hemagglutination-inhibition; ND, not determined.

†09SW64, A/swine/Ohio/09SW64/2009(H3N2); 10SW215, A/swine/Ohio/10SW215/2010(H3N2); 11SW347, A/swine/Ohio/11SW347/2011(H3N2); CIVH3N2, A/canine/Guangdong/1/2006(H3N2); CIVH3N8, A/canine/Iowa/13628/2005(H3N8); 99AIVH3N2, A/blue-winged teal/Ohio/99–31/99(H3N2); 11AIVH3N2, A/blue-winged teal/Ohio/11OS2474/2011(H3N2); Perth09, A/Perth/16/2009(H3N2); Victora11, A/Victoria/361/2011(H3N2); pdm09, A/California/7/2009(H1N1)

‡Values in bold are HI titers with homologous influenza isolates that were used to generate ferret antiserum. Each HI value in this table is an average number from 2 experiments.

Figure 1

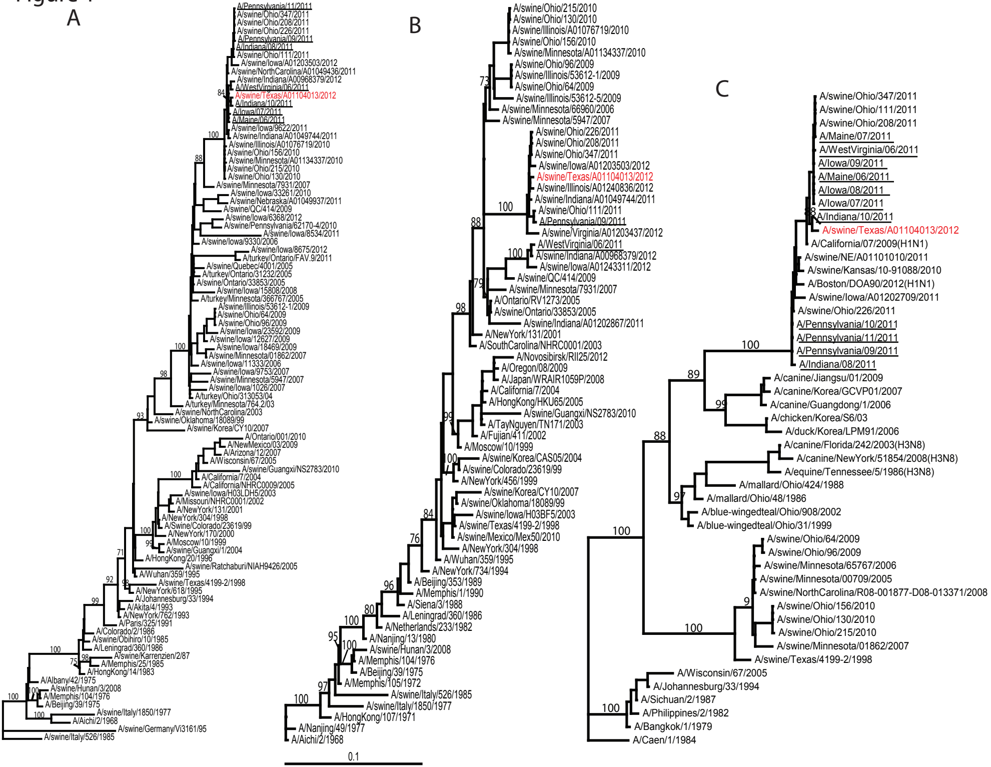

A
A/Pennsylvania/11/2011
A/swine/Ohio/347/2011
A/swine/Ohio/208/2011
A/swine/Ohio/226/2011
A/Pennsylvania/09/2011
A/Indiana/08/2011
A/swine/Ohio/111/2011
A/swine/Iowa/A01203503/2012
A/swine/NorthCarolina/A01049436/2011
A/swine/Indiana/A00968379/2012
A/WestVirginia/06/2011
A/swine/Texas/A01104013/2012
A/Indiana/10/2011
A/Iowa/07/2011
A/Maine/06/2011
A/swine/Iowa/9622/2011
A/swine/Indiana/A01049744/2011
A/swine/Illinois/A01076719/2010
A/swine/Ohio/156/2010
A/swine/Minnesota/A01134337/2010
A/swine/Ohio/215/2010
A/swine/Ohio/130/2010
A/swine/Minnesota/7931/2007
A/swine/Iowa/33261/2010
A/swine/Nebraska/A01049937/2011
A/swine/QC/414/2009
A/swine/Iowa/6368/2012
A/swine/Pennsylvania/62170-4/2010
A/swine/Iowa/8534/2011
A/swine/Iowa/9330/2006
A/swine/Iowa/8675/2012
A/turkey/Ontario/FAV.9/2011
A/swine/Quebec/4001/2005
A/turkey/Ontario/31232/2005
A/swine/Ontario/33853/2005
A/swine/Iowa/15808/2008
A/turkey/Minnesota/366767/2005
A/swine/Illinois/53612-1/2009
A/swine/Ohio/64/2009
A/swine/Ohio/96/2009
A/swine/Iowa/23592/2009
A/swine/Iowa/12627/2009
A/swine/Iowa/18469/2009
A/swine/Minnesota/01862/2007
A/swine/Iowa/11333/2006
A/swine/Iowa/9753/2007
A/swine/Minnesota/5947/2007
A/swine/Iowa/1026/2007
A/turkey/Ohio/313053/04
A/turkey/Minnesota/764.2/03
A/swine/NorthCarolina/2003
A/swine/Oklahoma/18089/99
A/swine/Korea/CY10/2007
A/Ontario/001/2010
A/NewMexico/03/2009
A/Arizona/12/2007
A/Wisconsin/67/2005
A/swine/Guangxi/NS2783/2010
A/California/7/2004
A/California/NHRC0009/2005
A/swine/Iowa/H03LDH5/2003
A/Missouri/NHRC0001/2002
A/NewYork/131/2001
A/NewYork/304/1998
A/Swine/Colorado/23619/99
A/NewYork/170/2000
A/Moscow/10/1999
A/swine/Guangxi/1/2004
A/HongKong/20/1996
A/swine/Ratchaburi/NIAH9426/2005
A/Wuhan/359/1995
A/swine/Texas/4199-2/1998
A/NewYork/618/1995
A/Johannesburg/33/1994
A/Akita/4/1993
A/NewYork/762/1993
A/Paris/325/1991
A/Colorado/2/1986
A/swine/Obihiro/10/1985
A/Leningrad/360/1986
A/swine/Karrenzien/2/87
A/Memphis/25/1985
A/HongKong/14/1983
A/Albany/42/1975
A/swine/Hunan/3/2008
A/Memphis/104/1976
A/Beijing/39/1975
A/swine/Italy/1850/1977
A/Aichi/2/1968
A/swine/Germany/Vi3161/95
A/swine/Italy/526/1985
84
100
88
100
98
93
100
100
99
71
92
98
99
100
75
98
100
100
100
B
A/swine/Ohio/215/2010
A/swine/Ohio/130/2010
A/swine/Illinois/A01076719/2010
A/swine/Ohio/156/2010
A/swine/Minnesota/A01134337/2010
A/swine/Ohio/96/2009
A/swine/Illinois/53612-1/2009
A/swine/Ohio/64/2009
A/swine/Illinois/53612-5/2009
A/swine/Minnesota/66960/2006
A/swine/Minnesota/5947/2007
A/swine/Ohio/226/2011
A/swine/Ohio/208/2011
A/swine/Ohio/347/2011
A/swine/Iowa/A01203503/2012
A/swine/Texas/A01104013/2012
A/swine/Illinois/A01240836/2012
A/swine/Indiana/A01049744/2012
A/swine/Ohio/111/2011
A/Pennsylvania/09/2011
A/swine/Virginia/A01203437/2012
A/WestVirginia/06/2011
A/swine/Indiana/A00968379/2012
A/swine/Iowa/A01243311/2012
A/swine/QC/414/2009
A/swine/Minnesota/7931/2007
A/Ontario/RV1273/2005
A/swine/Ontario/33853/2005
A/swine/Indiana/A01202867/2011
A/NewYork/131/2001
A/SouthCarolina/NHRC0001/2003
A/Novosibirsk/RII25/2012
A/Oregon/08/2009
A/Japan/WRAIR1059P/2008
A/California/7/2004
A/HongKong/HKU65/2005
A/swine/Guangxi/NS2783/2010
A/TayNguyen/TN171/2003
A/Fujian/411/2002
A/Moscow/10/1999
A/swine/Korea/CAS05/2004
A/swine/Colorado/23619/99
A/NewYork/456/1999
A/swine/Korea/CY10/2007
A/swine/Oklahoma/18089/99
A/swine/Iowa/H03BF5/2003
A/swine/Texas/4199-2/1998
A/swine/Mexico/Mex50/2010
A/NewYork/304/1998
A/Wuhan/359/1995
A/NewYork/734/1994
A/Beijing/353/1989
A/Memphis/1/1990
A/Siena/3/1988
A/Leningrad/360/1986
A/Netherlands/233/1982
A/Nanjing/13/1980
A/swine/Hunan/3/2008
A/Memphis/104/1976
A/Beijing/39/1975
A/Memphis/105/1972
A/swine/Italy/526/1985
A/swine/Italy/1850/1977
A/HongKong/107/1971
A/Nanjing/49/1977
A/Aichi/2/1968
73
100
88
100
79
98
99
100
84
76
96
80
100
95
100
97
100
0.1
C
A/swine/Ohio/347/2011
A/swine/Ohio/111/2011
A/swine/Ohio/208/2011
A/Maine/07/2011
A/WestVirginia/06/2011
A/Iowa/09/2011
A/Maine/06/2011
A/Iowa/08/2011
A/Iowa/07/2011
A/Indiana/10/2011
A/swine/Texas/A01104013/2012
A/California/07/2009(H1N1)
A/swine/NE/A01101010/2011
A/swine/Kansas/10-91088/2010
A/Boston/DOA90/2012(H1N1)
A/swine/Iowa/A01202709/2011
A/swine/Ohio/226/2011
A/Pennsylvania/10/2011
A/Pennsylvania/11/2011
A/Pennsylvania/09/2011
A/Indiana/08/2011
A/canine/Jiangsu/01/2009
A/canine/Korea/GCVP01/2007
A/canine/Guangdong/1/2006
A/chicken/Korea/S6/03
A/duck/Korea/LPM91/2006
A/canine/Florida/242/2003(H3N8)
A/canine/NewYork/51854/2008(H3N8)
A/equine/Tennessee/5/1986(H3N8)
A/mallard/Ohio/424/1988
A/mallard/Ohio/48/1986
A/blue-wingedteal/Ohio/908/2002
A/blue-wingedteal/Ohio/31/1999
A/swine/Ohio/64/2009
A/swine/Ohio/96/2009
A/swine/Minnesota/65767/2006
A/swine/Minnesota/00709/2005
A/swine/NorthCarolina/R08-001877-D08-013371/2008
A/swine/Ohio/156/2010
A/swine/Ohio/130/2010
A/swine/Ohio/215/2010
A/swine/Minnesota/01862/2007
A/swine/Texas/4199-2/1998
A/Wisconsin/67/2005
A/Johannesburg/33/1994
A/Sichuan/2/1987
A/Philippines/2/1982
A/Bangkok/1/1979
A/Caen/1/1984
88
100
89
99
88
97
100
9
100
100

Figure S1D PB2

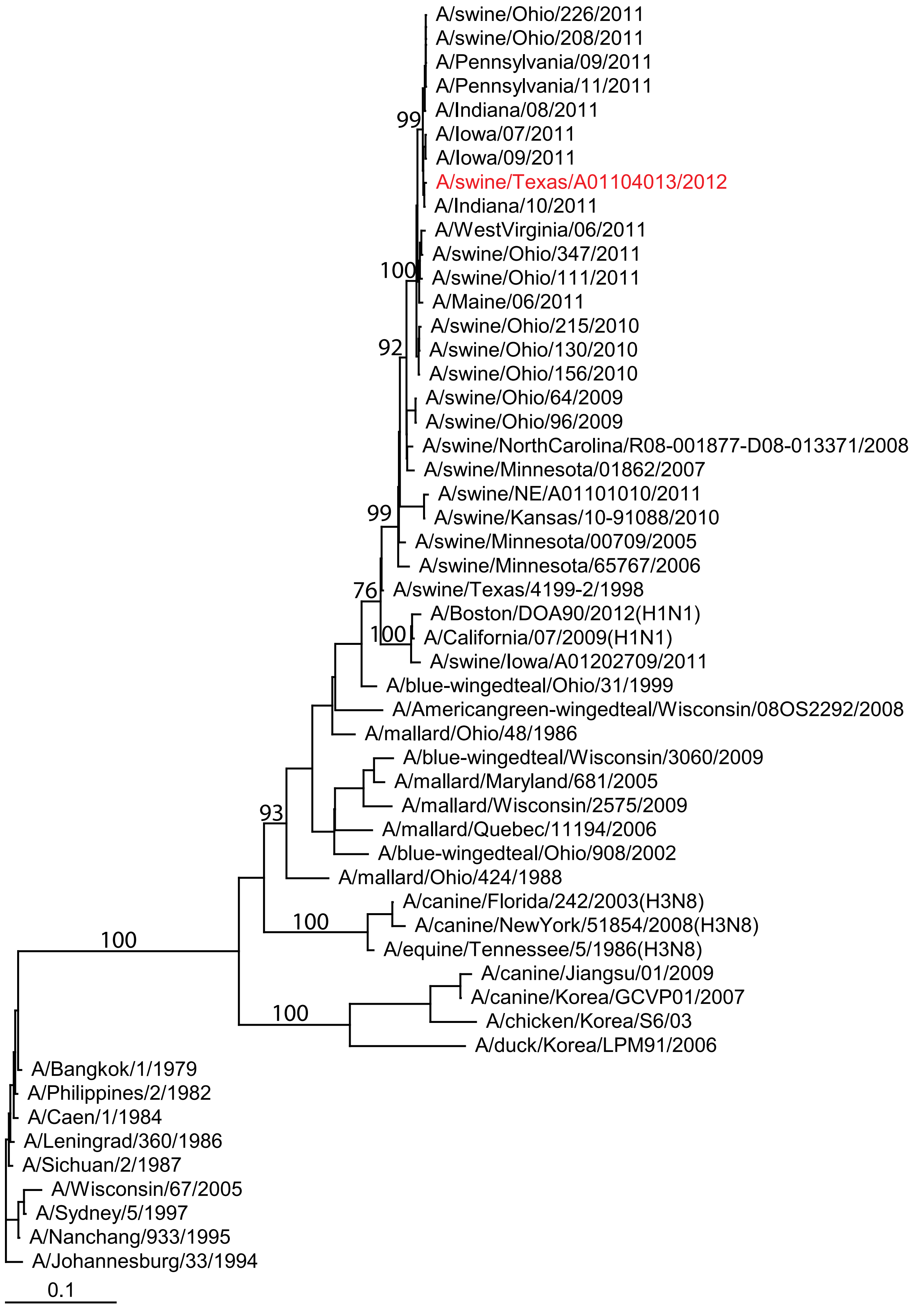

Figure S1E PB1

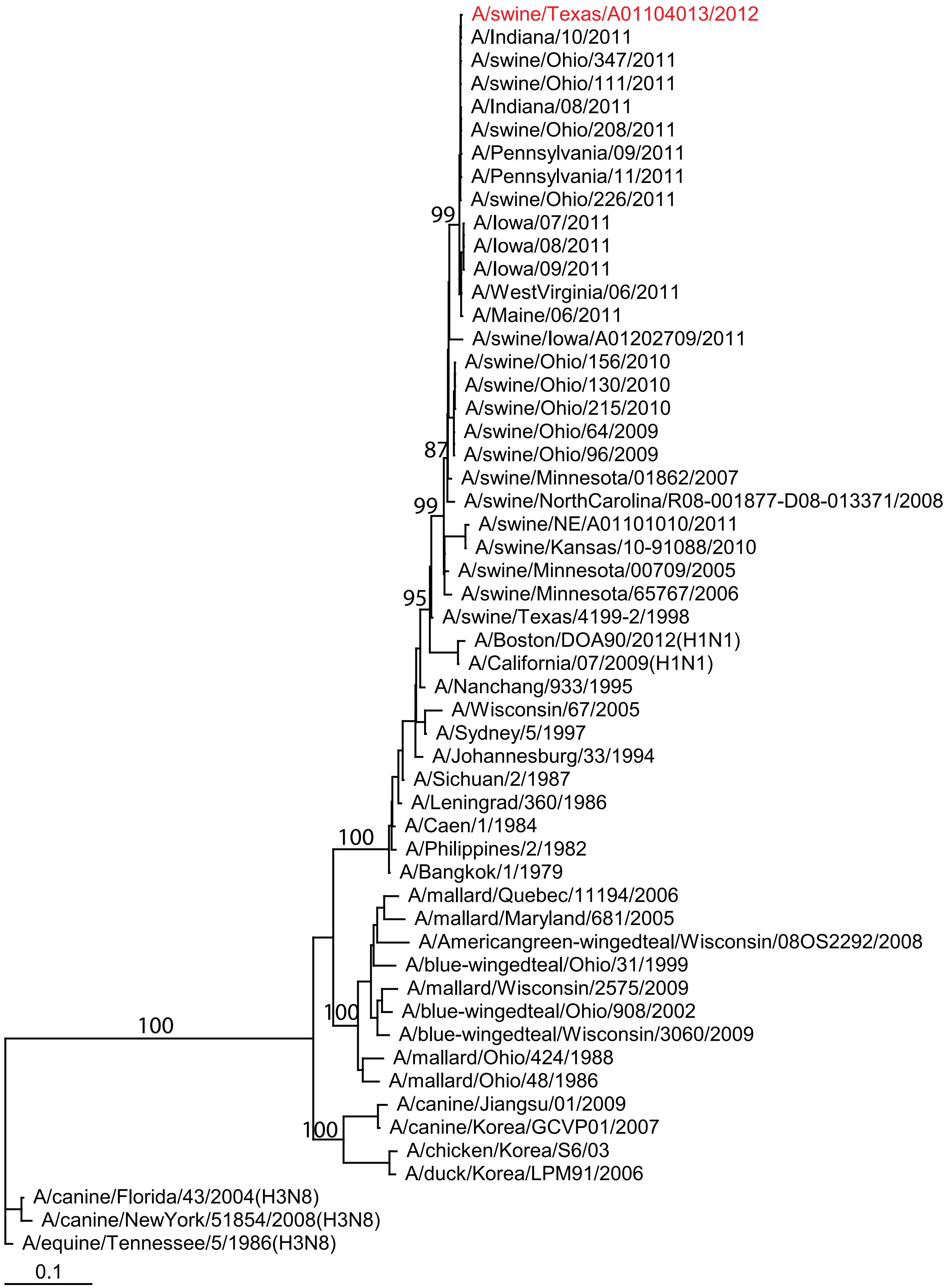

Figure S1F PA

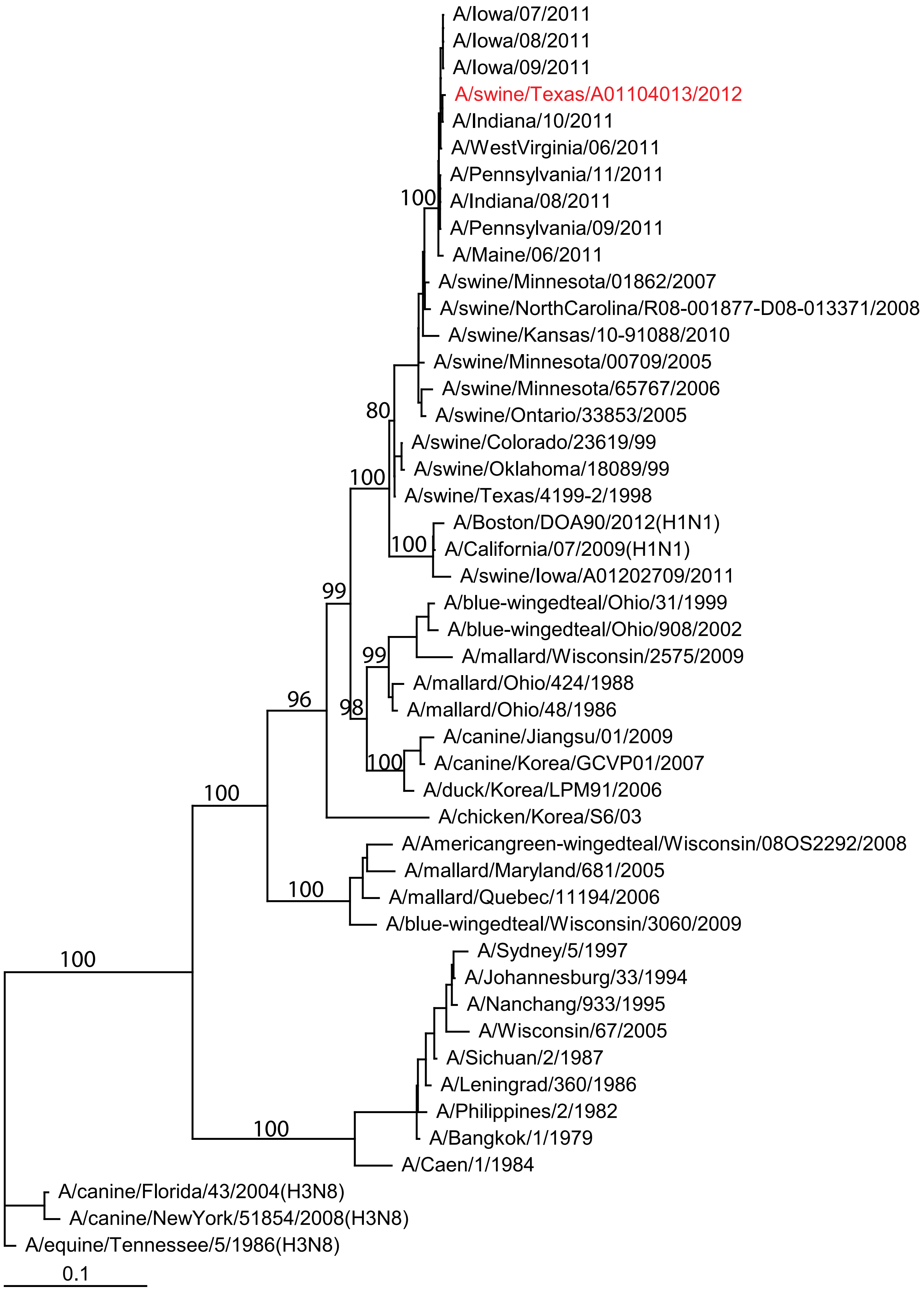

Figure S1G NP

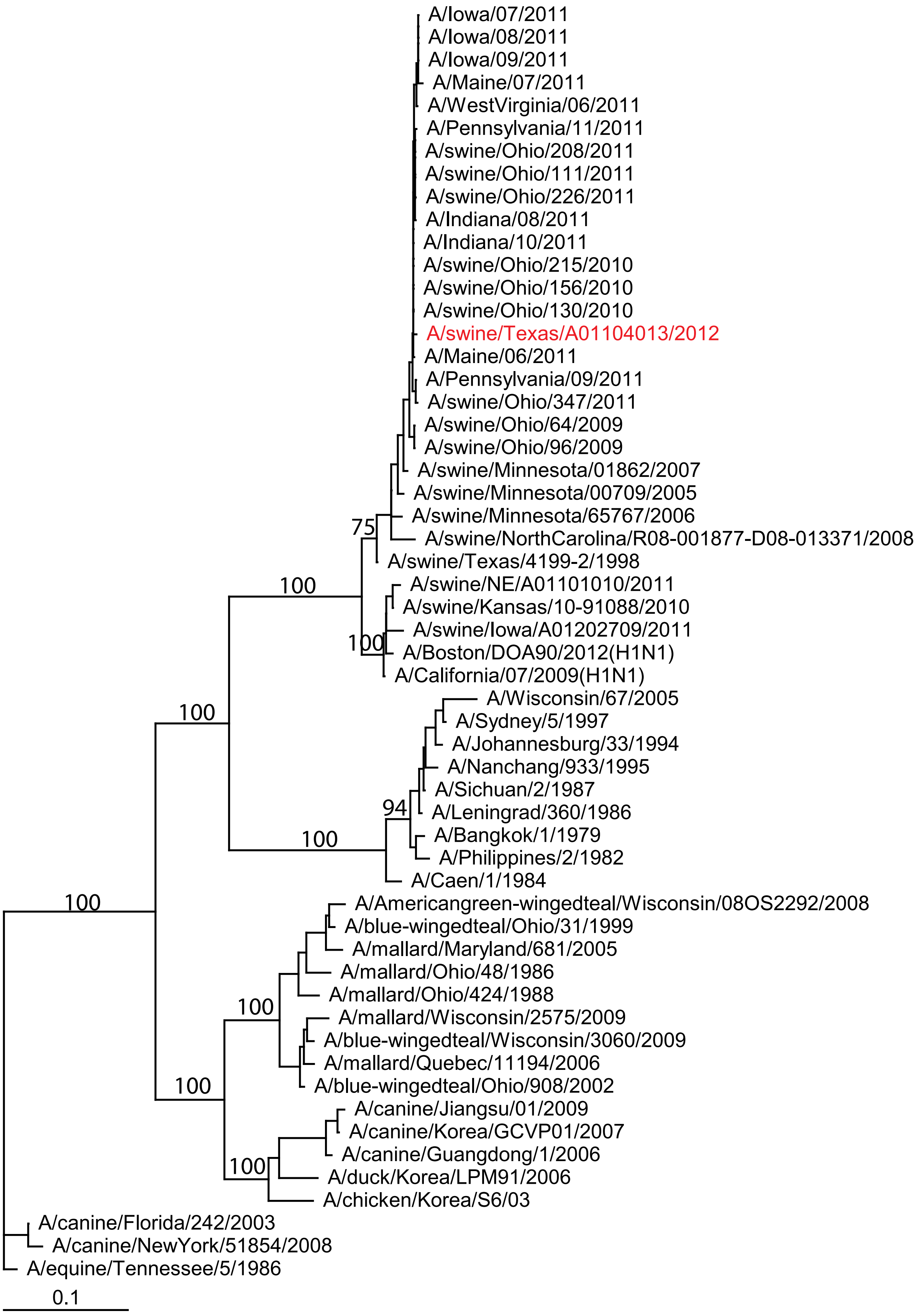

Figure S1H NS

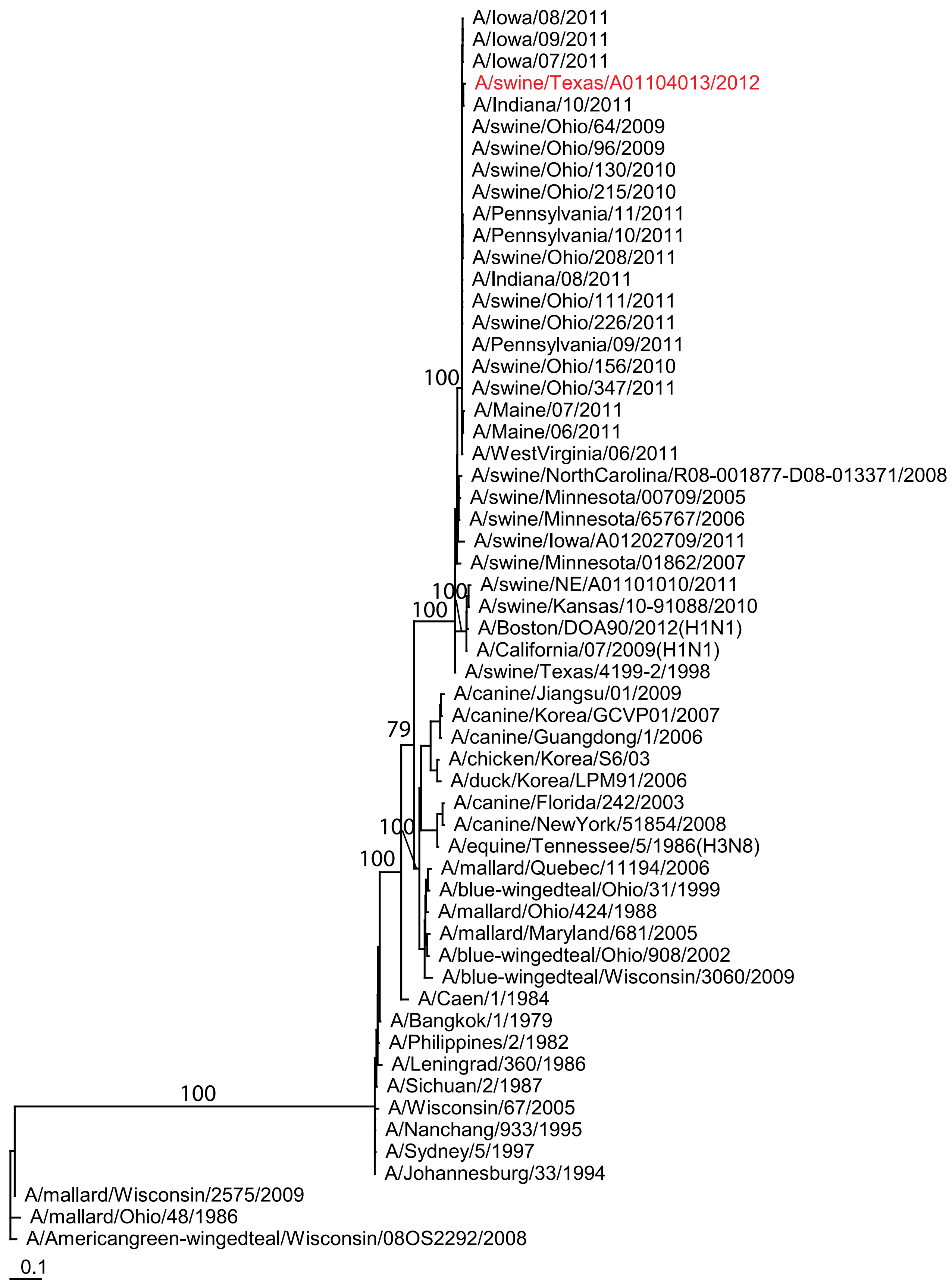

Technical Appendix Figure. Phylogenetic analysis of HA (A), NA (B), PB2 (C), PB1 (D), PA (E), NP (F), MP (G), and NS (H) gene segments of H3N2 influenza A virus recovered from feral swine. The H3N2 feral swine virus is marked in red, and human H3N2 variant (H3N2v) influenza virus isolates are underlined. The phylogenetic analyses were performed by using maximum-likelihood method as described (*8*). Sequence analyses showed that the HA, NA, PB2, PB1, PA, NP, MP, and NS genes of A/swine/Texas/A01104013/2012(H3N2) have 99.82%, 99.85%, 99.89%, 99.82%, 99.81%, 99.73%, 99.59%, and 99.40% nt sequence identity similar to those of A/Indiana/10/2011(H3N2), isolated from county fairs, respectively.
